# Role of HIF-1α/ERRα in Enhancing Cancer Cell Metabolism and Promoting Resistance of Endometrial Cancer Cells to Pyroptosis

**DOI:** 10.3389/fonc.2022.881252

**Published:** 2022-06-21

**Authors:** Pingping Su, Lirui Yu, Xiaodan Mao, Pengming Sun

**Affiliations:** ^1^ Laboratory of Gynecological Oncology, Department of Gynecology, Fujian Maternity and Child Health Hospital, Affiliated Hospital of Fujian Medical University, Fuzhou, China; ^2^ Fujian Key Laboratory of Women and Children’s Critical Diseases Research, Fuzhou, China

**Keywords:** glucose metabolism, lipid metabolism, HIF-1α, ERRα, endometrial cancer

## Abstract

Oxygen is critical to energy metabolism, and tumors are often characterized by a hypoxic microenvironment. Owing to the high metabolic energy demand of malignant tumor cells, their survival is promoted by metabolic reprogramming in the hypoxic microenvironment, which can confer tumor cell resistance to pyroptosis. Pyroptosis resistance can inhibit anti-tumor immunity and promote the development of malignant tumors. Hypoxia inducible factor-1α (HIF-1α) is a key regulator of metabolic reprogramming in tumor cells, and estrogen-related receptor α (ERRα) plays a key role in regulating cellular energy metabolism. Therefore, the close interaction between HIF-1α and ERRα influences the metabolic and functional changes in cancer cells. In this review, we summarize the reprogramming of tumor metabolism involving HIF-1α/ERRα. We review our understanding of the role of HIF-1α/ERRα in promoting tumor growth adaptation and pyroptosis resistance, emphasize its key role in energy homeostasis, and explore the regulation of HIF-1α/ERRα in preventing and/or treating endometrial carcinoma patients. This review provides a new perspective for the study of the molecular mechanisms of metabolic changes in tumor progression.

## Introduction

Endometrial carcinoma (EC) ranks first among malignant tumors of the female reproductive system in developed countries and second in China (1). Over the past decade, the EC morbidity and mortality rates have gradually increased (2), and the age of patients is younger than previously observed (3). According to the statistics of the National Cancer Center, in 2019, the incidence of EC in China was 10.28/100,000, and the mortality rate was 1.9/100000. The mortality rate of uterine cancer is twice as high as its morbidity rate (4). This may be due to the fact that specific pathological types of EC, such as serous carcinoma or EC with high risk factors (such as distant metastasis or deep myometrial invasion), are resistant to pyroptosis after radiotherapy/chemotherapy, resulting in the risk of tumor recurrence and metastasis, and a significantly increased mortality rate (5). Although recent progress has been made in the diagnosis and treatment of EC, and better clinical outcomes have been achieved, the quality of life of patients is affected by the side effects of treatment. These can include infertility, surgical menopause, lower limb lymphedema, sexual dysfunction, depression, and fatigue, which pose a serious threat to women’s health (6, 7).

In 1983, Bokhman (8) classified EC into type I and type II according to the presence or absence of estrogen stimulation. The diagnosis and prognosis of type I EC are related to mutations in the genes *PTEN*, *PI3KCA*, *POLE*, *CTNNB1*, and *TP53*; deletions of the DNA mismatch repair protein; and the expression of estrogen receptor and progesterone receptor. The prognosis of type II EC is related to the overexpression of human epidermal growth factor receptor 2 (HER2) and mutations in the *TP53* gene. The activation of PI3K/Akt, P53, mitogen-activated protein kinase, and Wnt/β-catenin signaling pathways is closely related to the pathogenesis of EC (9). In 2013, a molecular genetic analysis of 373 EC patient samples was carried out based on the Cancer Genome Atlas (TCGA), and cases were divided into four subgroups as follows: POLE mutation, microsatellite-instable (MSI), copy number low (CNL), and copy number high (CNH). Among them, patients with POLE mutations have the best prognosis, those with MSI and CNL have a moderate prognosis, and those with the CNH-mutant type have the worst prognosis (10). Therefore, it is necessary to better understand the molecular changes in the progression of EC and develop new biomarkers or targeted therapy to further improve the overall prognosis of patients with EC.

Despite the obvious heterogeneity of EC, only the standardized treatment is still used for all subtypes. Since the Bokhman classification cannot explain the high morphological and molecular heterogeneity of EC, the histopathological classification method based on tumor morphology and tumor grade needs to be further improved. Santoro et al. (11) proposed the distribution and prognosis of different histopathological types of EC in a molecular classification based on TCGA, put forward the improvement in risk stratification based on molecules, and discussed the corresponding treatment methods as well. These observations need to be evaluated by experienced gynecologic oncologists, pathologists, and geneticists. Histopathology and TCGA molecular typing combined with diagnosis and treatment methods can be used to accurately treat EC and predict the susceptibility and prognosis of EC to specific therapeutic drugs (12). However, according to the current diagnosis and treatment guidelines and the medical situation, it is impossible to perform a detailed molecular typing in every EC patient. This requires a multi-disciplinary team (MDT) (molecular biologists, clinicians, oncologists, geneticists, and other professionals, as well as paramedical units, such as pathology, ultrasound, and imaging) to decide the treatment method that is most likely to benefit the patients, by integrating medical history, complications, clinical, histomorphology, immunohistochemistry and molecular data. Through cross-cooperation in the MDT, a dynamic assessment of the condition, treatment, and regular reexamination can be carried out in the form of joint and inter-disciplinary consultations, so that the diagnosis and treatment strategy can be adjusted in time and a more reasonable treatment plan can be provided. MDT mode can be applied to the whole process of prevention, diagnosis, preoperative evaluation, postoperative treatment, and follow-up of EC outpatient service, which can improve the quality of life and prognosis of cancer patients (13).

**Figure 1:**
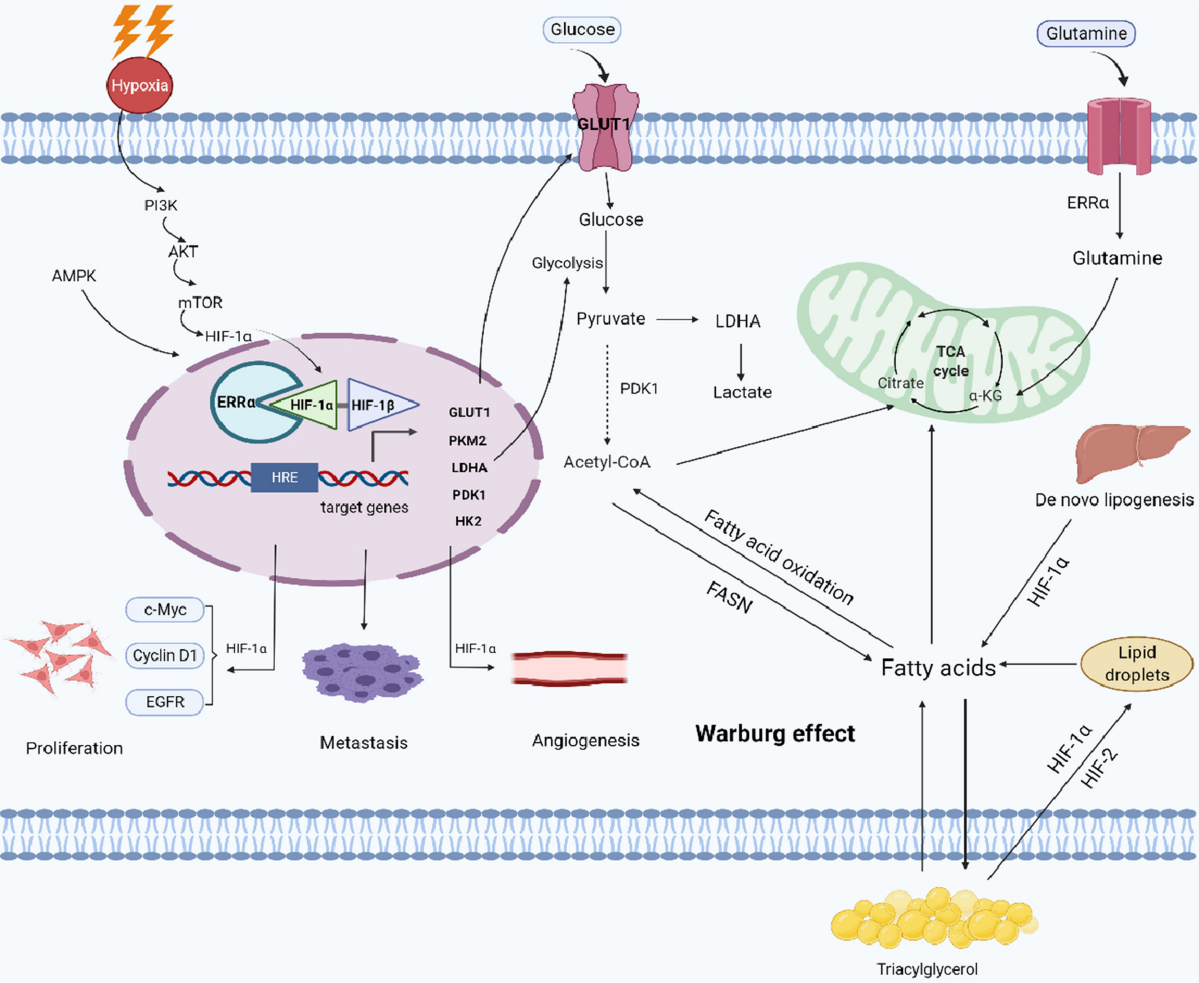
Working model of the role of HIF-1α/ERRα in cancer cell metabolism. Cancer cells can adapt to low oxygen conditions through the PI3K/AKT/mTOR and AMPK signaling pathways, which regulate metabolic reprogramming. ERRα interacts with HIF-1α, enhancing the transcriptional activity of HIF-1α and promoting the remodeling of glucose and lipid metabolism (formation of lipid droplets) in cancer cells. ERRα also enhances glutamine metabolism and lipid *de novo* synthesis, promoting metabolic adaptation in cancer cells. Together, these activities stimulate tumor proliferation, metastasis, and angiogenesis. Images were made in BioRender (Biorender.com).

Hypertension, obesity, and diabetes are the triple risk factors of EC; in fact, EC can be regarded as a metabolic disease (14). Inflammation may be activated in EC patients due to oxidative stress and increased systemic inflammation caused by metabolic syndrome, and tumor cells reprogram their metabolic pathways in this inflammatory environment to maintain a higher proliferation rate; promote tumor growth, invasion, and neovascularization; and resist cell death signals (15–17). Hypoxia inducible factor-1α (HIF-1α) is a key regulator of tumor invasion and a key promoter of energy adaptation. Increased HIF-1α activity promotes angiogenesis, metabolic reprogramming, metabolic adaptation, extracellular matrix remodeling, epithelial mesenchymal transformation (EMT), invasion, metastasis, resistance to radiotherapy and chemotherapy, maintenance of cancer stem cell phenotype, immune escape, and protein expression affecting changes in the tumor immune microenvironment during early carcinogenesis (18, 19). HIF-1α is a prognostic marker related to the overall poor prognosis of tumor patients (20). Estrogen-related receptor α (ERRα) subtypes are highly expressed in malignant tumors, especially in hormone-dependent/related tumors, such as breast cancer (BRCA) (21), prostate cancer (PCa) (22), ovarian cancer (23), and endometrial cancer (24–26). At the same time, ERRα, acting as a transcription factor, regulates the process of energy metabolism in the body *via* the tricarboxylic acid cycle (27), oxidative phosphorylation (OXPHOS) (28), and glucose and lipid metabolism (29). As a result, a variety of biological effects are observed, such as tumor cell proliferation, differentiation, and apoptosis; tumor angiogenesis; and control of systemic inflammation. These processes are related to the prognosis and progression of the tumor. At present, clarifying how tumor cells resist pyroptosis and adapt to hypoxia through metabolic reprogramming, is a hot research topic aimed at overcoming metastatic/recurrent tumors using anti-metabolic therapy. We hypothesized that metabolic reprogramming intervention of tumor cells may be an important approach for the treatment of metastatic tumors. Based on this hypothesis, this review summarizes the role of metabolic changes caused by HIF-1α/ERRα in the metabolic reprogramming of tumor cells in the growth, invasion, and metastasis of EC (**Figure 1**). These areas are very important for understanding the occurrence, progression, and treatment stratification of EC.

## HIF-1α Participates in Regulated Glucose and Lipid Metabolism in Cancer Cells

The metabolic phenotype of cancer cells varies in different types of cancer; for example, some malignant tumors mainly rely on glycolysis, whereas others exhibit an OXPHOS-mediated phenotype (30). In general, coordinated catabolism and anabolism are essential for tumor cells to maintain an energy supply and biosynthesis (31). The metabolic adaptation process of tumors is driven by several key carcinogenic signal cascades or kinase signals, including specific genes such as *cMYC* (32, 33), adenosine monophosphate-activated protein kinase (AMPK) (34), and PI3K/Akt/mTOR (35). *Myc* directly activates the transcription of glycolytic enzymes, namely *GLUT1*, *LDHA*, *PKM2*, and *HK2*; coordinates cell metabolism and cell proliferation; and promotes cell malignant transformation (32, 33). These key rate-limiting enzymes involved in glycolysis promote the development of drug resistance in tumor cells, which proves the importance and effectiveness of targeted therapy in the regulation of energy metabolism in malignant tumor cells. AMPK restores the cellular ATP pool by promoting catabolism and inhibiting anabolism and plays a central role in regulating the reprogramming of cellular energy metabolism to adapt to metabolic stress (36, 37). Inhibition of the PI3K/AKT/mTOR/HIF-1α signaling pathway can reduce the expression of the key glycolytic enzymes *PKM2* and *LDHA*, thus inducing cell death and improving the cytotoxicity of cisplatin (38). *PTEN* can inhibit the activity of PI3K/AKT/mTOR pathway, and *PTEN* dysfunction is the most common genetic change in EC (39). Therefore, targeting PI3K may improve the personalized treatment of EC patients with *PTEN* mutation (40). Energy for tumor cells is provided by the high activity of aerobic glycolysis stimulated by HIF-1α. This creates an acidic microenvironment that promotes EMT and leads to more invasive phenotypes, such as radiotherapy and chemotherapy resistance or tumor metastasis, which are closely related to poor clinical prognosis (41). It is suggested that the regulation of HIF-1α may be a new approach to overcome the drug resistance of tumor cells. Hence, further exploration of the malignant progression of tumors driven by HIF-1α through the above carcinogenic pathway will contribute to the development of better treatment methodologies for tumors.

The reprogramming of lipid metabolism has become a marker of malignant tumors (42, 43). Lipids make up the basic components of the cell plasma membrane and they are also important signal molecules and energy sources. Lipid biosynthesis plays an important role in highly proliferative malignant tumor cells (44) (such as prostate, breast, liver, and kidney cancers) supporting the production of cell membrane and the regulation of its fluidity; promoting the formation of triacylglycerides and energy storage; promoting resistance to reactive oxygen species (ROS) damage (45, 46); and producing signal molecules involved in cancer cell migration, inflammation, and survival (47). These changes promote the further development of cancer. HIF-1α-dependent changes in lipid metabolism can promote the survival and growth of cancer cells. Lipid droplets can promote cancer cell proliferation and tumor growth, and there is a positive correlation between the level of lipid droplets in tumors and a poor prognosis (48). The accumulation of lipid droplets has been confirmed in different types of cancers, including breast (49, 50), cervical (51), prostate (52), and ovarian (53) cancers. Under hypoxic conditions, because oxygen-dependent stearoyl-CoA desaturase is inhibited (54), the ratio of saturated/unsaturated fatty acids changes, thus affecting the integrity of cell membranes and cell function. HIF-1α inhibits the accumulation of saturated lipids and reduces the toxicity induced by saturated fatty acids by promoting the expression of fatty acid synthase, triggering fatty acid synthesis and activation, which promote the production of unsaturated fatty acids (55, 56). These findings emphasize the importance of changes in lipid metabolism in promoting tumor development and suggest that targeting lipid metabolism in tumor cells may be a new approach for the treatment of patients with malignant tumors (**Table 1**). However, there are still few studies on the effects of HIF-1α on the distribution of lipid metabolism and the reprogramming of lipid metabolism in tumor cells.

**Table 1:** Studies on HIF-1α regulation of glucose and lipid metabolism and promotion of gynecological malignant tumor progression.

Author	Cancer type	Main finding
Giatromanolaki et al. (57)	EC	HIF-1α is highly expressed in proliferating endometrium nuclei, which is related to tumor invasion.
Wincewicz et al. (58)	EC	Activators of transcription (*STAT3*) mediates the signal transduction of HIF-1α to stimulate tumor growth and maintain the invasive ability of EC cells.
Seeber et al. (59)	EC	HIF-1α expression is often accompanied by the activation of its downstream factor *GLUT-1*, which makes cancer cells survive in the hypoxic environment, and is related to aggressive tumor behavior.
Yeramian et al. (60)	EC	HIF-1α regulates the transcriptional activity of NF- κB and the accumulation of nuclear RelA in Ishikawa cells, and mediates the survival of EC cells under hypoxia.
Ai et al. (61)	Ovarian cancer	Knockout of HIF-1α can redirect aerobic glycolysis in drug-resistant ovarian cancer cells to mitochondrial OXPHOS, resulting in cell death through the production of ROS, thus improving the response of cisplatin-resistant ovarian cancer cells to cisplatin.
Triantafyllou et al. (62)	Cervical cancer	In tumor hypoxia microenvironments, HIF-1α promotes fatty acid uptake by inducing fatty acid binding protein and PPARγ, inducing phosphatidic acid phosphatase *LIPIN1* production, regulating fatty acid synthesis, and promoting lipid storage by regulating the expression of acylglycerol-3-phosphate acyltransferase 2 (*AGPAT2*) and *LIPIN1*. This results in chemotherapy resistance in tumor cells.
Gong et al. (63)	EC	AGR2, a member of the endoplasmic reticulum resident protein disulfide isomerase family, induces lactate dehydrogenase A (*LDHA*), phosphoglycerate kinase 1 (*PGK1*), kallikrein 2(*HK2*), and enolase 1-α(*ENO1*) expression, glucose uptake, and lactic acid production as well as promotes the progress of EC through the MUC1/HIF-1α pathway.
Gao et al. (64)	Ovarian cancer	As it is a key regulator of glucose metabolism in ovarian cancer cells, activation of the PI3K/AKT/HIF-1α signaling pathway plays an important carcinogenic role in promoting the growth and metastasis of ovarian cancer.

## ERRα Participates in Regulated Glucose and Lipid Metabolism in Cancer Cells

ERRα can control key metabolic processes by transcriptional regulation of different metabolic genes during cell differentiation (65). ERRα inhibits glucose oxidation by transcriptionally activating pyruvate dehydrogenase kinase 4 (*PDK4*) (66). Inhibition of ERRα can block mitochondrial respiration and enhance the effect of tumor chemotherapy (67). HIF-1α can improve oxygen-dependent energy metabolism through mitochondrial biogenesis, mainly through the signal transduction pathway of the PGC-1α/HIF-1α/ERRα axis. This, in turn, promotes the reprogramming of tumor metabolism and leads to tumor metastasis and progression. Therefore, we infer that ERRα is a key regulator of metabolic reprogramming under glucose stress and hypoxia.

ERRα plays an important role as a nuclear transcription factor in lipid metabolism. Increased activity of ERRα can increase the oxidation rate of fatty acids. Genetic or pharmacological inhibition of ERRα can reduce fat weight and lipid accumulation and resist high fat-diet-induced obesity (68). ERRα can also stimulate adipogenesis by increasing the accumulation of triglycerides (69). From a metabolic standpoint, lipid metabolism is significantly upregulated in EC, affecting the treatment outcome and/or disease progression of patients (70). The ERRα axis is a central regulator of metabolism in malignant tumors, especially in BRCA (71). High levels of ERRα activity maximize energy production by promoting mitochondrial metabolism and angiogenesis, thus meeting the energy metabolic needs of rapid cell proliferation (72, 73). These observations provide a causal relationship to explain how ERRα regulates tumor metabolism (**Table 2**).

**Table 2:** Studies on ERRα regulation of glucose and lipid metabolism and promotion of hromone-related tumor progression.

Author	Cancer type	Main finding
Fujimoto et al. (74)	EC	ERRα binds to steroid receptor coactivator family without any ligand, drives the transcription activity of target gene, and inhibits estrogen response element-dependent transcription activity in the presence of estrogen, which is related to the growth and progress of EC.
McGuirk et al. (75)	BRCA	After using lapatinib to inhibit receptor tyrosine kinase, BRCA cells increased glutamine metabolism and lipid *de novo* synthesis, while reducing ROS production through the PGC-1α/ERRα axis, promoting cell metabolic adaptation.
Deblois et al. (76)	BRCA	ERRα triggers the adaptive change of mitochondrial energy metabolism in drug-resistant cells by increasing glutamine metabolism and detoxification of active oxygen required for cell survival under the condition of therapeutic stress, which leads to lapatinib resistance in BRCA.
Zou et al. (77)	PCa	ERRα can cooperate with HIF-1α to regulate angiogenesis and glycolysis, thus promoting the growth of tumor cells under hypoxia
Matsushima et al. (78)	EC	SiRNA-ERRα inhibits VEGF and cell proliferation and induces cell cycle arrest during mitosis followed by apoptosis through caspase-3 signal.
Park et al. (79)	BRCA	ERRα antagonist destroys mitochondrial function, inhibits lactic acid utilization, damages the activity of cancer cells, and increases the activity of PI3K/mTOR inhibitor.
Audet-Walsh et al. (80)	BRCA	PGC-1α/ERRα axis, as an inhibitor of folate cycle metabolism and purine biosynthesis, targets PGC-1α/ERRα to make BRCA cells sensitive to folate treatment.
Huang et al. (81)	EC	ERRα directly binds to the promoter of TGFB1, thus increasing its transcription and triggering the migration and invasion of EC cells.
Sun et al. (82)	EC	Down-regulation of ERRα can inhibit TFEB, which is mediated by PGC1α and participates in the mTOR signal pathway. In addition, under the mediation of Tcf, down-regulation of ERRα can increase the expression of Oct3/4 and participate in the Wnt signaling pathway.
Kokabu et al. (83)	EC	ERRα may play a role in the upstream of Akt and/or regulate the Akt/mTOR signaling pathway in EC. XCT790 significantly inhibits tumor growth and angiogenesis *in vivo* and induces cell apoptosis.
Mao et al. (84)	EC	TAM combined with XCT790 can promote the proliferation inhibition and apoptosis of EC endothelial cells when targeting ERα and ERRα.
Yoriki et al. (85)	EC	ERRα inhibits the TGF-β-induced EC metastasis through tumor-stromal interaction.
Park et al. (86)	BRCA	ERRα inhibition interferes with pyruvate transport into mitochondria by inhibiting the expression of mitochondrial pyruvate carrier 1, revealing that the NADPH generation pathway is a therapeutic direction for BRCA.
Chen et al. (26)	EC	Overexpression of ERRα increases the expression of PGC-1 α and the activity of TFEB in EC cells and promotes EMT.
Huang et al. (18)	EC	As a potential agonist of PPARγ, ERRα inhibitor promotes cell proliferation and inhibits apoptosis through the Bcl-2/Caspase3 pathway in EC.
Schoepke et al. (87)	PCa	As a selective ERRα/γ inverse agonist, SLU-PP-1072 can inhibit the Warburg effect, change the metabolism and gene expression of PCa cells, and lead to cell cycle disorder and apoptosis.
Brindisi et al. (88)	BRCA	Cholesterol and mevalonate are related to the progression, invasiveness, and drug resistance of BRCA by activating the ERRα pathway.
Casaburi et al. (89)	BRCA	Cholesterol has been identified as a natural ERRα ligand. High cholesterol content and ERRα activity can promote ERRα-mediated proliferation of BRCA cells and expression of metabolic target genes by producing different cytokines, thus contributing to the inflammatory environment.
Li et al. (90)	BRCA	ERRα enhances the resistance of BRCA to lapatinib by targeting the region of SHMT2 promoter and activating transcription and then regulating the metabolic adaptability of mitochondria.
Casaburi et al. (91)	BRCA	Cholesterol promotes ERRα-mediated metabolic target gene expression, and increases NADPH level and cell proliferation.

## HIF-1α and ERRα Crosstalk in Cancer, Especially in Endometrial Carcinoma

Metabolic reprogramming is essential for the survival of cancer cells in hypoxia and nutrient deficient environments. The surviving cancer cells have higher metabolic plasticity (92) and show more malignant biological behaviors such as invasiveness and chemotherapy resistance. The glycolytic activity of EC cells is considerably higher than that of non-tumor cells and contributes to the progression of EC (93). Therefore, it is very important to screen metabolic genes related to the progression of EC and select targeted treatments to improve the prognosis of EC patients. Jiang et al. (94) used The Cancer Genome Atlas database to analyze the expression of metabolism-related genes (MRGs). In this way, they could screen for differentially expressed MRGs (DE-MRGs) significantly related to the prognosis of EC patients. Functional enrichment analysis of DE-MRGs showed that most of these MRGs were enriched in amino acid, glycolysis, and glycerol phospholipid metabolism.

It is known that HIF-1α regulates a large group of genes/proteins involved in cell metabolism, pH, and EMT, thus making tumor cells more aggressive than that of other phenotypes (95). Hypoxia is related to angiogenesis in EC in which the transition from metabolism to aerobic glycolysis (Warburg effect) and tumor cell resistance to pyroptosis are very important for tumor growth (96). EC patients with tumors highly expressing HIF-1α exhibit a decreased disease-free survival. The expression of HIF-1α is an important prognostic factor in patients with EC and is related to myometrial invasion, histological grade, and recurrence after radiotherapy (59, 60). However, at present, no studies have examined the effect of differences in HIF-1α expression in relation to the different molecular types of EC. Furthermore, the exact mechanism underlying the role of HIF-1α in the malignant progression of EC is not clear. Byrne et al. (97) also found that glycolysis and lipogenesis are highly related to the malignant phenotype of EC. Inhibition of *GLUT6* expression can inhibit glycolysis and the survival of EC cells, which reflects the key role of energy metabolism in tumor progression.

ERR is composed of ERRα, ERRβ, and ERRγ and is referred to as the central regulator of energy metabolism; its natural ligand has not been determined (98). An imbalance in ERRα activity can significantly affect cell metabolic homeostasis, causing metabolic disorders and cancer. In 2006, Sun et al. proposed that an imbalance in ERRα expression may be an important reason for the carcinogenesis of EC (99). Subsequently, when a lentiviral vector overexpressing ERRα was constructed and transfected into EC cells, the proliferation of EC cells was promoted (84, 100). XCT790 can effectively inhibit the expression of ERRα in EC. The expression of ERRα is closely related to the proliferation and apoptosis of EC cells (82). In 2018, it was proposed that ERRα is a key regulator of cell metabolism and plays an important role in gynecological endocrine-related tumors and energy metabolism (101). A knockout of ERRα can inhibit the invasion, metastasis, and angiogenesis of EC and promote apoptosis (83, 102). Endogenous or exogenous inhibition of the expression and function of ERRα has obvious anticancer effects (82). ERRα is also expressed in BRCA, PCa, ovarian cancer, and other malignant tumors, and the increase in ERRα expression is positively correlated with the late progression of malignant tumors (103), indicating that ERRα plays a regulatory role in tumor growth. Xia et al. (104) used *in vivo* and *in vitro* experiments to regulate the activity of ERRα. Through integration of gene expression profiles and genome-wide chromatin immunoprecipitation techniques, they determined the key role of ERRα in lipid and carbohydrate metabolism and mitochondrial function under physiological and pathological conditions. Furthermore, they were able to clarify the targeting of ERRα as a new way to treat metabolic disorders and other related diseases. Although a high expression level of ERRα in EC indicates a poor prognosis, the specific mechanism of the role of ERRα in the progression of EC has not been elucidated.

Functional studies in BRCA cells showed that ERRα can promote cancer growth through a variety of transcriptional regulatory networks or mechanisms, including enhancing HIF-1-dependent hypoxic cell growth (86), activating vascular endothelial growth factor promoting tumor angiogenesis (105), and enhancing glycolysis producing the Warburg effect (79). Studies by Zou et al. (77) showed that the regulation of carcinogenicity by ERRα in PCa is a key hypoxic growth regulator and an important cofactor of HIF-1α in hypoxic microenvironments. ERRα enhances HIF-1α signal transduction by interacting with HIF-1α, which makes ERRα-overexpressing PCa cells better adapted to a hypoxic microenvironment.

The activation of ERRs involves genes related to mitochondrial biogenesis and OXPHOS. However, this enhanced mitochondrial oxidation capacity is mainly targeted at fatty-acid metabolism because ERRs upregulate PDK4 preventing glucose oxidation. Since HIF-1α can activate PDK1 (106), it is essential for the transformation from glucose oxidation *via* the tricarboxylic acid cycle to glycolytic metabolism under hypoxic conditions. This shared function of inhibiting glucose respiratory metabolism may represent the internal relationship between ERRα and HIF-1α. The change in glucose and lipid metabolism is an important feature of the occurrence and development of EC. Combined with the regulation of glucose and lipid metabolism by ERRα and HIF-1α, the regulation of HIF-1α/ERRα may be a means to inhibit malignant progression, such as invasion and metastasis, of EC.

## The Involvement of Pyroptosis in Endometrial Carcinoma

Cell resistance to pyroptosis is very important for tumor growth. Pyroptosis is widely involved in the occurrence and development of tumors, infectious diseases, metabolic diseases, nervous system-related diseases, and atherosclerotic diseases, but its specific regulatory mechanism is not clear (107). NOD-like receptor family, pyrin domain-containing protein 3 (NLRP3) activation of inflammatory bodies can activate caspase-1, which in turn, mediates the conversion of gasdermin D (GSDMD) and pro-IL-1β into active forms. GSDMD is a key effector of pyroptosis, forming pores in the plasma membrane that eventually leads to cell expansion and membrane dissolution. Pyroptosis resistance can inhibit anti-tumor immunity and promote the development of many types of cancers. Pyroptosis induces the production of inflammatory factors, such as IL-1 β, which act as alarm signals to activate and recruit immune cells to mediate the immune response. The upregulation of GSDMD protein induces the infiltration of M1 macrophages, CD4+, CD8+T lymphocytes, and other immune cells, and increases the sensitivity of cells to anti-PD-1 monoclonal antibody (108).

EC patients with *POLE* mutations and MSI exhibited high expression of PD-1 and PD-L1, accompanied by a large number of tumor-infiltrating lymphocytes, which indicates their suitability for immunotherapy (109). Liang et al. (110) found pyroptosis-related lncRNAs in TCGA database, which can be used to predict the prognosis and suitability for immunotherapy in EC. Pyroptosis-related genes in EC, such as GSDMD, are involved in the Wnt signaling and the substance metabolism pathways (111). Pyroptosis is closely related to the proliferation, invasion, and metastasis of cancer cells and can affect the efficacy of chemotherapy (112). However, the mechanisms by which tumor cells promote pyroptosis resistance by remodeling the energy metabolism need more exploration and confirmation, both *in vivo* and *in vitro*.

At present, there are various targeted therapies for EC. For example, anti-angiogenic drugs, such as bevacizumab, combined with vascular endothelial growth factor (VEGF), are used to inhibit tumor growth and metastasis (113). Abnormal activation of the PI3K/AKt/mTOR pathway is related to tumor metabolism, cell growth, invasion and migration, and angiogenesis. As a rapamycin analogue, ridaforolimus can effectively inhibit mTOR, thus inhibiting tumor growth and improving the progression-free survival of EC patients (114, 115). Immunotherapy, such as the pembrolizumab anti-PD-1 monoclonal antibody, can inhibit tumor progression (116). Some scholars have suggested that tumors with a mutation in the *POLE* gene can exhibit the differential regulation of tumor cell metabolism through glucose metabolism (117). Targeted therapy for EC provides a new treatment direction for advanced and recurrent EC. The clinical effects of chemotherapy in EC patients are different. Chemotherapy resistance is mediated by bypassing pyroptosis (108). The inhibition of ERRα enhances the chemotherapeutic effect in tumors (67). However, whether ERRα inhibition can promote chemotherapy resistance in tumor cells by blocking mitochondrial respiration, increasing ROS production, and then inducing cell pyroptosis needs further research. By adding targeted therapy, especially targeting a specific cell metabolic process, to chemotherapy, the survival of EC can be improved.

**Figure 2:**
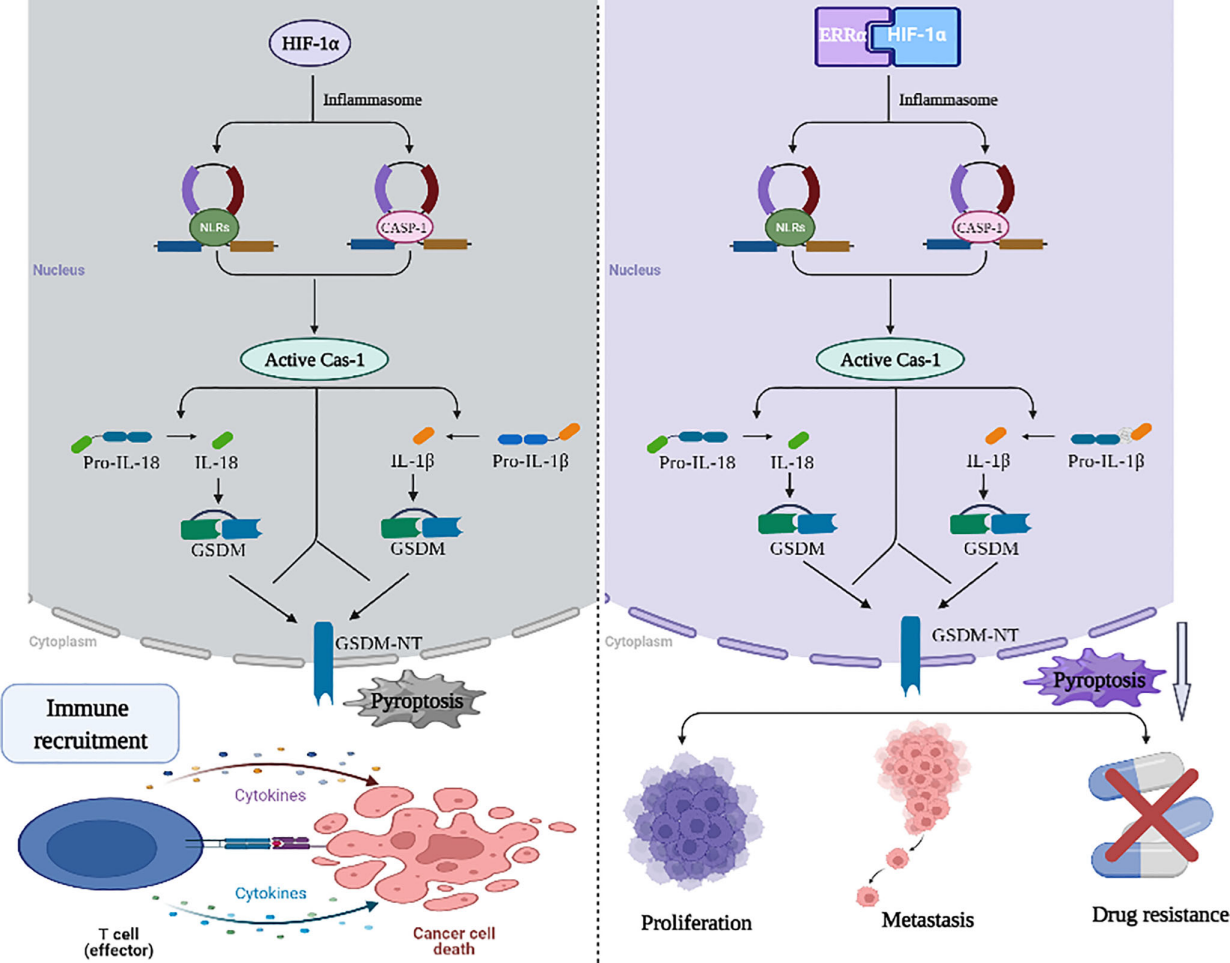
Working model of the role of HIF-1α/ERRα in pyroptosis resistance. HIF-1 α is a component of the molecular mechanism of pyroptosis mediated by NLRP3 inflammatory bodies, which can recruit immune cells to mediate the immune response. HIF-1α/ERRα interaction promotes the adaptation of tumor cells to hypoxia and promotes cancer cell resistance to pyroptosis. These activities stimulate tumor proliferation, metastasis, and drug resistance. Images were made in BioRender (Biorender.com).

Hypoxia can lead to malignant progression of tumor cells through the following mechanisms: inhibiting the activity of immune cells, providing energy support for tumor cells, improving immune resistance of tumor cells, inhibiting apoptosis of tumor cells, and promoting metastasis of tumor cells (118, 119). ERRα cooperates with HIF-1α to regulate angiogenesis and glycolysis, thus promoting the growth of tumor cells under hypoxia, while stable HIF-1α further increases the expression of ERRα at the transcriptional level (77). HIF-1α/ERRα interaction promotes the adaptation of tumor cells to hypoxia, suggesting that there is a positive circuit among HIF-1α, ERRα, and mitochondrial biogenesis. However, in regard to the hypoxic microenvironment formed by the rapid proliferation of tumor cells, there have been studies on the effects of HIF-1α/ERRα interactions with glycolysis and angiogenesis. Whether EC cells with high expression of ERRα show enhanced anti-pyroptosis properties through interactions with HIF-1α, or whether they can show an anti-pyroptosis effect independent of HIF-1α, remains to be further studied. The effect of HIF-1α and ERRα on tumor microenvironment remodeling and chemotherapy resistance in EC is not clear, and their interaction may be an important mechanism for EC cells to resist pyroptosis (**Figure 2**).

## Conclusion

EC is a gynecological disease accompanied by metabolic impairment, which requires management to improve the glucose and lipid metabolism of the patients, in addition to cancer treatment. Complications in EC patients can be resolved through a combined treatment strategy with inputs from gynecological oncology, nutrition, endocrinology, cardiology, and other disciplines. Tumor cells metabolically adapt to their microenvironment through metabolic reprogramming to meet their energy needs for proliferation and differentiation. Metabolic dysfunction in the cancer microenvironment can cause different outcomes. MRGs can be used as prognostic markers of tumors. Inhibition of hypoxia-induced metabolic pathways may be a new and promising therapeutic strategy. In this review, we presented evidence for the regulatory effect of HIF-1α and ERRα on MRGs through various signaling pathways in malignant tumors, thus changing the energy metabolism of tumor cells and causing tumor cell resistance to pyroptosis, clarifying the role of key genes in metabolic pathways. With continued exploration of natural endogenous ERRα ligands and understanding of the regulation of HIF-1α, the key goal in the future is to develop drugs that regulate the transcriptional activity of HIF-1α/ERRα to prevent and treat metabolism-related malignant tumors and other diseases. A future challenge for cancer researchers is to transform basic research into clinical application by designing clinical trials based on the molecular characteristics of EC, to study novel drugs for targeted monotherapy, or in combination with existing cytotoxic drugs.

## Author Contributions

PPS carried out the primary literature search, drafted and revised the manuscript, tables, figures, and participated in discussions. PMS conceived the idea and edited the manuscript. LY helped edited the figures. XM helped modify the manuscript. All authors have read and agreed to the published version of the manuscript.

## Funding

This research was supported by the Fund of Natural ScienceFoundation of Fujian Province, grant number 2020J02059 and 2021J01404, and Joint Funds for the Innovation of Science and Technology, Fujian Province (Grant no. 2020Y9160).

## Conflict of Interest

The authors declare that the research was conducted in the absence of any commercial or financial relationships that could be construed as a potential conflict of interest.

## Publisher’s Note

All claims expressed in this article are solely those of the authors and do not necessarily represent those of their affiliated organizations, or those of the publisher, the editors and the reviewers. Any product that may be evaluated in this article, or claim that may be made by its manufacturer, is not guaranteed or endorsed by the publisher.

## Abbreviations

HIF-1α, Hypoxia inducible factor-1α; ERRα, Estrogen-related receptors α; EC, Endometrial carcinoma; MSI, Microsatellite-instable, CNL, Copy number low; CNH, Copy number high; EMT, Epithelial mesenchymal transformation; BRCA, Breast cancer; PCa, Prostate cancer; OXPHOS, Oxidative phosphorylation; NSCLC, Non-small cell lung cancer cells; EGFR, Epidermal growth factor receptor; HCC, Hepatocellular carcinoma; LDHA, Lactate dehydrogenase A; PGK1, Phosphoglycerate kinase 1; HK2, Kallikrein 2; ENO1, Enolase 1-α; PDK4, Pyruvate dehydrogenase kinase 4; PGC-1α, Peroxisome proliferator-activated receptor gamma coactivator 1-alpha; ROS, Reactive oxygen species metabolism-related genes; DE-MRGs, Differentially expressed metabolism-related genes; FABP, Fatty Acid Binding Protein; NLRP3, NOD-like receptor family, pyrin domain-containing protein 3; GSDMD, Gasdermin D; VEGF, Vascular endothelial growth factor.
